# Spatial differentiation and integrated tourism development of China’s traditional heritage villages

**DOI:** 10.3389/fsoc.2026.1803562

**Published:** 2026-07-15

**Authors:** Chaohui Zheng, Heng Wei

**Affiliations:** 1 Faculty of Science and Engineering, University of Nottingham, Nottingham, United Kingdom; 2 College of Urban and Rural Construction, Zhongkai University of Agriculture and Engineering, Guangzhou, China

**Keywords:** K-means, rural cultural heritage, rural integrated tourism, rural tourism development, traditional village

## Abstract

**Introduction:**

Traditional villages in China serve as important carriers of rural cultural heritage but are increasingly threatened by rapid urbanisation and uneven regional development. Understanding their spatial distribution and integration within cultural–tourism networks is essential for developing effective conservation and regional planning strategies.

**Methods:**

Using a national dataset of 8,157 officially designated traditional villages, combined with city-level tourism indicators and three categories of cultural heritage carriers (A-level scenic spots, intangible cultural heritage, and protected cultural relics), this study developed an integrated analytical framework based on spatial statistical analysis, spatial correlation analysis, and K-means clustering to evaluate spatial differentiation and cultural-tourism integration patterns.

**Results:**

The results reveal a pronounced “south-dense, north-sparse” spatial distribution with persistent regional inequality. Traditional villages exhibit weak correlations with overall tourism performance but strong spatial coupling with A-level scenic spots, moderate associations with protected cultural relics, and limited integration with intangible cultural heritage, indicating a selective and uneven embedding structure. Based on these relationships, Chinese cities can be classified into four development types: comprehensive leading areas, scenic–village integration areas, balanced development areas, and potential development areas, each characterised by distinct cultural–tourism integration pathways.

**Discussion:**

The findings demonstrate that traditional villages function as selectively embedded nodes within China’s cultural–tourism network rather than uniformly integrated tourism resources. This typology provides a practical basis for differentiated conservation policies, cultural heritage management, and place-based tourism planning while offering new insights into the spatial organisation of cultural heritage systems at the national scale.

## 1 Introduction

Traditional villages in China represent a unique form of rural cultural heritage that embodies centuries of architectural tradition, settlement morphology, agricultural practice, and intangible cultural expression (Qian et al., 2025). As carriers of vernacular building techniques, local belief systems, clan structures, and region-specific landscapes, these villages serve as living archives of historical rural life and social organization (Nursanty et al., 2024). Since 2012, the Chinese government has launched a nationwide identification and protection programme, recognizing six batches comprising 8,157 traditional villages and establishing the largest coordinated rural heritage conservation effort in the world (Jing et al., 2024). This initiative reflects growing awareness that traditional villages are not only cultural resources but also critical components of rural revitalization, ecological civilization construction, and the preservation of cultural diversity in the context of rapid societal transformation. However, accelerated urbanization (Zerbe, 2022), demographic decline (Atreya et al., 2018), land consolidation (Zhou et al., 2020), and modernization pressures (Liu et al., 2010) continue to threaten the survival of these settlements, resulting in the disappearance of village clusters, the fragmentation of cultural landscapes, and the erosion of traditional knowledge systems (Zeng et al., 2023). Similar challenges have been documented globally across rural heritage sites, where development-driven interventions frequently disrupt historical continuity and undermine community-based cultural practices (Egusquiza et al., 2021). Against this backdrop, safeguarding traditional villages has become an urgent national and international priority, positioning them at the intersection of cultural heritage conservation, sustainable rural development, and integrated regional planning.

Yet despite their recognized importance, how traditional villages are spatially differentiated at the national scale and embedded within China’s cultural–tourism systems remains insufficiently understood. This study therefore pursues two core objectives. The first is to generate a comprehensive, data-driven evaluation of the spatial differentiation of traditional villages across China, with particular attention to the regional disparities and clustering mechanisms that shape their national distribution. The second is to examine the structural relationship between traditional villages and urban tourism systems by assessing their associations with tourism development levels and key cultural heritage resources. These objectives establish an integrated analytical framework that extends beyond case-based research and situates traditional villages within broader spatial, cultural, and economic processes. Accordingly, the study is guided by three research questions: (1) What spatial differentiation patterns characterize traditional villages at the national scale, and how do indicators such as the Nearest Neighbor Index, Geographical Concentration Index, and Gini-based inequality measures capture their clustering, dispersion, and polarization? (2) What is the nature of the relationship between traditional villages and urban tourism development, and to what extent do village distributions correspond with indicators such as tourist arrivals and tourism revenue? (3) How are traditional villages spatially associated with major cultural–tourism carriers including A-level scenic spots, intangible cultural heritage items, and nationally protected heritage sites and can these associations be used to identify distinct integration types?

Although spatial differentiation is central to understanding the distribution of traditional villages, spatial patterns alone cannot explain how these villages are embedded in contemporary cultural and tourism systems. Examining their relationships with A-level scenic spots (Qiu et al., 2021), intangible cultural heritage items (Xue and Huang, 2025) and protected cultural relics (Zhang et al., 2025) is therefore essential to determine whether traditional villages function as isolated heritage units or as components of larger heritage clusters (Huang et al., 2024). On this basis, a clustering approach is employed to classify major tourism cities according to their integration profiles, providing a comparative framework for identifying regions with strong, moderate or limited capacity for coordinated heritage–tourism development. In doing so, the study contributes a national-scale quantitative examination of the spatial differentiation of traditional villages, addressing a gap in a literature that is predominantly local in scope, and advances empirical understanding of their functional positioning within broader cultural–tourism systems. The proposed clustering framework also offers an operational basis for comparing integration patterns across cities and supports the formulation of differentiated conservation and tourism planning strategies.

## 2 Literature review

### 2.1 Spatial distribution of traditional villages

Despite growing scholarly interest in rural heritage conservation, existing research on traditional villages in China remains heavily fragmented and predominantly localized (Wu et al., 2020). Most studies focus on individual provinces (Chen et al., 2025), specific cultural regions (Zhou et al., 2022), or case-based heritage assessments (Hu et al., 2019), resulting in limited understanding of how traditional villages are spatially organized across the national landscape. Zhu et al. (2023) applied ArcGIS spatial analysis, geographic statistical methods, and historical interpretation to reveal that traditional villages in Xiangxi exhibit a clustered distribution with a northeastward evolutionary shift, driven jointly by natural, socio-economic, and cultural factors. Liu et al. (2022) used NNI, kernel density estimation, standard deviation ellipse, and location entropy to show that intangible cultural heritage along the ancient Qin–Shu roads exhibits strong spatial agglomeration, a northeast–southwest temporal shift, and is shaped jointly by natural conditions, transportation, traditional villages, and demographic evolution. Zhang et al. (2021) applied remote sensing and GIS analysis to map the spatial distribution and morphological evolution of silo-cave villages on the Loess Plateau, revealing their concentration in economically underdeveloped regions and identifying three distinct transformation patterns shaped by landforms, transport accessibility, economic conditions, and demographic change. Consequently, the broader spatial logics such as the pronounced “dense-southeast, sparse-northwest” distribution pattern remain insufficiently quantified and inadequately explained.

At the national scale, a small but growing body of work has begun to characterise the aggregate spatial properties of China’s traditional villages using the full multi-batch official dataset. Several studies have applied spatial statistics including nearest neighbour indices, kernel density estimation, and Gini-based inequality measures to demonstrate the pronounced “dense-southeast, sparse-northwest” distributional pattern and to quantify how inter-provincial polarisation evolved across successive designation rounds (Bian et al., 2022). Others have examined the driving forces behind this national pattern, identifying terrain complexity, ethnic minority settlement traditions, and historical agricultural continuity as primary explanatory factors, while noting that policy cycles and administrative capacity also mediate spatial outcomes (Chen et al., 2023).

### 2.2 Traditional villages and cultural-tourism integration

Alongside this spatial literature, a parallel body of research has investigated the relationship between traditional village conservation and cultural tourism development. Tourism has been widely recognised as a dual instrument for rural heritage: it can generate community income, incentivise conservation investment, and reinforce local cultural identity, yet poorly governed tourism development risks commodifying heritage assets and accelerating the erosion of authenticity (Gao and Wu, 2017). At regional and county scales, empirical studies have shown that villages with richer cultural–tourism resource endowments and greater proximity to high-level scenic areas and transport networks tend to achieve stronger tourism attractiveness and closer integration with surrounding tourist destinations (Dong et al., 2025; Xia et al., 2025). More recently, (Li et al., 2023) examined the spatial correlation and formation mechanisms between traditional villages and rural tourism in Henan Province, confirming that proximity to scenic areas and transport corridors significantly enhances tourism integration potential. Despite these contributions, the existing literature has not systematically evaluated the structural embedding of traditional villages within the cultural–tourism networks of major cities, nor has it quantified the associations between village distributions and formal cultural carriers such as A-level scenic spots, intangible cultural heritage items, and nationally protected cultural relics at the national scale.

Moreover, while cultural tourism has been widely promoted as a strategy for rural revitalization, few studies have systematically examined whether traditional villages are functionally or structurally integrated into the tourism systems of major cities (Yanan et al., 2024). The relationship between village heritage resources and urban tourism indicators, as well as the extent to which traditional villages contribute to regional tourism networks, has rarely been evaluated at a national scale (Tang et al., 2023). Equally understudied is the spatial association between traditional villages and key cultural–tourism carriers, including A-level scenic spots, intangible cultural heritage items, and nationally protected heritage sites (Shen et al., 2025). These gaps limit the ability to understand how traditional villages participate in contemporary cultural economies and constrain the development of coordinated urban–rural tourism strategies.

Taken together, existing studies provide a detailed account of where traditional villages are located, yet a much thinner understanding of how they are embedded within urban cultural–tourism systems. Addressing this imbalance requires a national-scale, integrated analysis, which the following section operationalises.

## 3 Research materials and methodology

### 3.1 Study area and data sources

**Figure 1:**
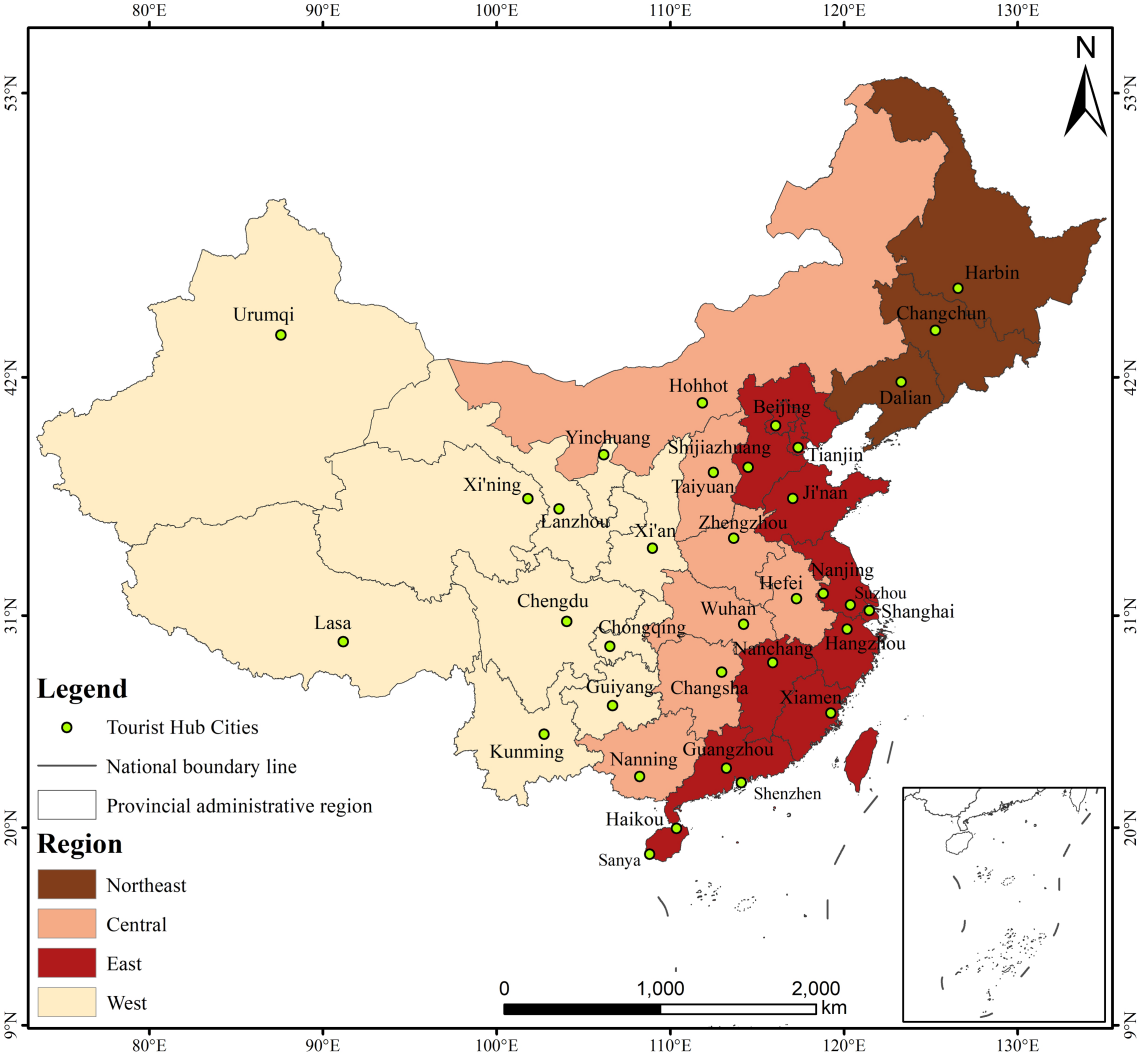
Research area.

The study area covers mainland China, with a specific analytical focus on the spatial distribution of nationally recognized traditional villages and their relationship with major tourist cities. Traditional villages represent historically preserved rural settlements officially identified by the Ministry of Housing and Urban–Rural Development (MOHURD) across six batches since 2012. In addition to the national traditional village dataset, the study incorporates 34 major tourist cities, including provincial capitals and key tourism-oriented prefecture-level cities. These cities serve as important nodes within China’s tourism system, exhibiting varying levels of tourism demand, tourism infrastructure development, and cultural heritage resources (see Figure 1).

These 34 cities were selected based on three criteria: first, official designation as a major tourist destination at the national or provincial level; second, availability of complete and standardised city-level tourism statistics including domestic tourist arrivals, tourism revenue, and inbound visitor data from the China Culture and Tourism Statistical Yearbook; and third, broad geographical coverage across China’s four major regions(Northeast, East, Central, and West) to ensure representativeness of the national urban tourism system. It should be noted that for municipalities directly under the central government (Beijing, Shanghai, Tianjin, and Chongqing), the number of traditional villages is calculated based on the full administrative area rather than the delineated urban tourism study area. This adjustment is made because these municipalities encompass extensive rural and mountainous regions, and restricting the analysis to core urban areas would significantly underestimate the actual distribution of traditional villages.

This study integrates three main categories of data: (1) Traditional Village Dataset: Data on the six batches of national traditional villages were obtained from the official lists released by MOHURD. The dataset includes village names, administrative locations, and geographic coordinates. In total, 8,157 villages were used, covering all 31 provinces and autonomous regions. (2) Tourism statistics of major cities: Urban-level tourism indicators were collected from the latest China Culture and Tourism Statistical Yearbook, official municipal statistical bulletins, and tourism bureau reports. (3) Geospatial Data: Administrative boundary data (national, provincial, and municipal levels) were obtained from the National Geomatics Center of China (NGCC). Geographic coordinates of traditional villages were geocoded and corrected using ArcGIS Pro. All datasets were standardized and spatially aligned within the WGS 84 coordinate system. Tourism indicators were normalized to eliminate scale differences prior to correlation and clustering analyses. Geocoding accuracy was manually checked to ensure reliable spatial representation of village locations.

### 3.2 Methods

**Figure 2:**
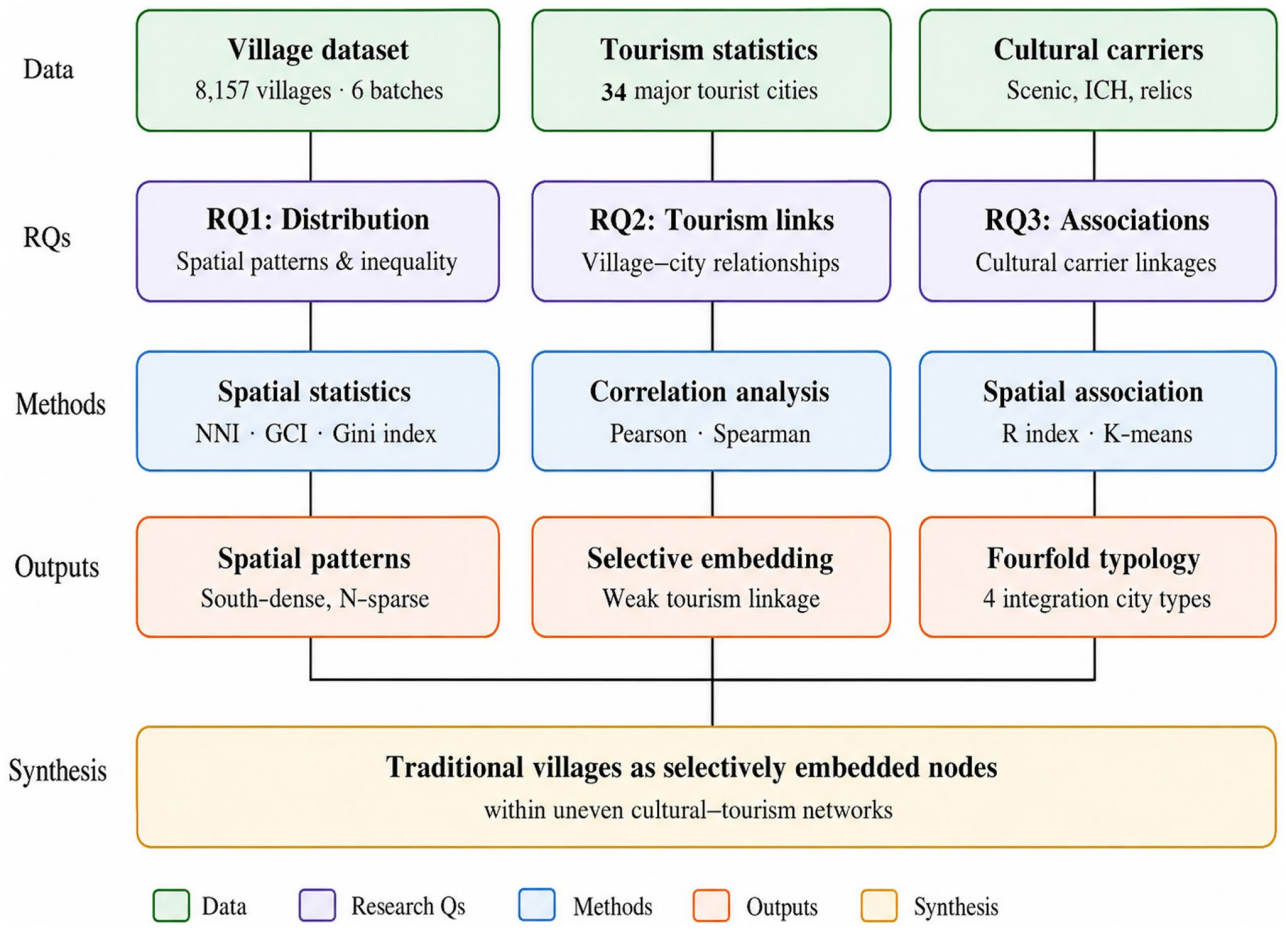
Research flow.

To clarify the analytical logic of the study, Figure 2 presents the overall methodological framework. The analysis is structured around three research questions, linking multiple datasets with a series of complementary analytical methods. Spatial statistical techniques are used to examine distribution patterns and regional disparities, correlation analysis is applied to assess relationships with tourism development, and clustering methods are employed to identify typological differences in integration. This integrated approach ensures a coherent connection between research questions, analytical procedures, and research findings.

#### 3.2.1 Nearest neighbor index and variation coefficient

The spatial pattern of traditional villages was first examined using the Nearest Neighbor Index (NNI), which characterizes whether the observed point distribution tends toward clustering, randomness, or dispersion (Lee et al., 2021). The index is defined as Equation 1:


$$\mathrm{NNI}=\frac{{\bar{d}}_{o}}{{\bar{d}}_{e}}$$ (1)

Where ${\bar{d}}_{o}=\frac{1}{n}\sum_{i=1}^{n}\;d_{i}$ represents the observed mean nearest-neighbor distance among the n villages, and ${\bar{d}}_{e}=\frac{1}{2\sqrt{\frac{n}{A}}}$ denotes the expected mean distance of a randomly distributed point pattern within area A. An NNI value less than 1 suggests a clustered distribution, a value close to 1 indicates randomness, and a value greater than 1 reflects a dispersed pattern. To further assess the degree of spatial imbalance among administrative units, the coefficient of variation (CV) was calculated as CV=σ/μ, , where σ and μ denote the standard deviation and mean of the number of traditional villages per region, respectively. Higher CV values indicate stronger spatial heterogeneity, revealing unequal distributional tendencies across regions.

#### 3.2.2 Geographical concentration index

The Geographical Concentration Index (GCI) was employed to evaluate the degree to which traditional villages are concentrated within particular provinces or metropolitan areas (Bi et al., 2024). The index is expressed as Equation 2:


$$G=\sum_{i=1}^{n}\;{\left(\frac{X_{i}}{T}\right)}^{2}$$ (2)

Where $X_{i}$ denotes the number of traditional villages in region i , T is the national total, and n is the number of regions. The value of G increases as the spatial distribution becomes more uneven, approaching 1 when villages are highly concentrated in only a few regions and approaching 1/n when evenly distributed.

#### 3.2.3 Inequality index

To quantify interregional disparities in village distribution, an Inequality Index based on the Gini coefficient was applied (Li et al., 2015). The coefficient is computed from cumulative population shares and cumulative village shares according to Equation 3:


$$G=1-\sum_{i=1}^{n}(Y_{i}+Y_{i-1})(X_{i}-X_{i-1})$$ (3)

Where $X_{i}$ and $Y_{i}$ represent the cumulative proportions of regions and traditional villages, respectively, after sorting regions by increasing village numbers. As an alternative and mathematically equivalent formulation, the Gini coefficient can also be expressed as Equation 4:


$$G=\frac{1}{2n^{2}\mu }\sum_{i=1}^{n}\;\sum_{j=1}^{n}|x_{i}-x_{j}|$$ (4)

with $x_{i}$ denoting the number of villages in region i and μ the national mean. Higher values of the inequality index indicate stronger spatial polarization and more pronounced regional imbalance. Conventional interpretive thresholds provide a meaningful framework for evaluating the spatial disparities reflected in the data.

#### 3.2.4 Pearson and Spearman correlation analysis

To quantitatively assess the association between the number of traditional villages and tourism development across major tourist cities, Pearson’s product–moment correlation coefficient and Spearman’s rank correlation coefficient were applied (Alsaqr, 2021). Let Y denote the number of traditional villages in each city, and X1 to X5 represent five tourism indicators: inbound tourist arrivals (X1), domestic tourist arrivals (X2), international tourism foreign-exchange earnings (X3), domestic tourism revenue (X4), and the proportion of tourism income in GDP (X5). Pearson’s correlation coefficient r measures the strength of linear association between variables and is expressed as Equation 5:


$$r=\frac{\sum_{i=1}^{n}(X_{i}-\bar{X})(Y_{i}-\bar{Y})}{\sqrt{\sum_{i=1}^{n}\;{\left(X_{i}-\bar{X}\right)}^{2}}\sqrt{\sum_{i=1}^{n}\;{\left(Y_{i}-\bar{Y}\right)}^{2}}}$$ (5)

Where, $X_{i}$ means tourism indicator value of the i -th city and $Y_{i}$ means the number of traditional villages in the i -th city. The $\bar{X}$ and $\bar{Y}$ represents the mean values of the corresponding variables and n represents the number of cities. The coefficient r ranges from −1 to 1, where values close to ±1 indicate strong linear association, and values near 0 indicate weak or no linear relationship.

To capture monotonic but potentially non-linear relationships, Spearman’s rank correlation coefficient ρ was also computed. After converting all variables into ranks, ρ is calculated as Equation 6:


$$\rho =1-\frac{6\sum d_{i}^{2}}{n(n^{2}-1)}$$ (6)

where $d_{i}$ means the difference between the ranks of $X_{i}$ and $Y_{i}$ , and n represents the number of observations. Spearman’s ρ is less sensitive to extreme values and does not require the assumption of normality, providing a robustness check for correlation patterns derived from Pearson’s coefficient.

#### 3.2.5 Spatial relationship model and spatial correlation index

To examine the spatial association between traditional villages and different cultural–tourism carriers, including A-level scenic spots, intangible cultural heritage (ICH), and nationally protected cultural relics, a spatial relationship model was adopted.

Following previous studies, a regular grid system was constructed across mainland China. Considering the typical spatial influence range of traditional villages and cultural–tourism carriers, a point diameter of 5 km was adopted and the grid spacing was set to four times the point diameter (20 km). Equal-distance grid lines were generated at 20 km intervals along both latitude and longitude directions, producing a series of spatial samples with an approximate area of 400 km^2^ per grid cell. Grid cells located along national boundaries were allowed to vary slightly in area.

The spatial relationship between traditional villages and cultural–tourism carriers was evaluated using a contingency-based spatial association model. Following the spatial overlay procedure, each grid cell was classified according to the co-occurrence pattern of traditional villages and the examined carrier, generating a four-cell contingency matrix. The resulting frequencies were used to calculate a spatial correlation index ( R ), which measures the degree of spatial association between the two distributions (Equation 7):


$$R=\frac{\mathrm{ad}-\mathrm{bc}}{\sqrt{\left(a+b)(c+d)(a+c)(b+d\right)}}$$ (7)

Where a denotes the number of grid cells containing both traditional villages and the examined carrier, b and c represent cells containing only one of the two elements, and d represents cells containing neither element. Values approaching 1 indicate strong positive spatial association, values near 0 indicate spatial independence, and negative values indicate spatial separation.

The statistical significance of the spatial association was evaluated using a chi-square test (Equation 8):


$$\chi^{2}=\frac{N{\left(\mathrm{ad}-\mathrm{bc}\right)}^{2}}{\left(a+b)(c+d)(a+c)(b+d\right)}$$ (8)

Where ( N ) denotes the total number of spatial samples. Following the chi-square distribution with one degree of freedom, the spatial association was considered statistically significant when ( $\chi^{2}$ > 3.841) at the 5% significance level.

#### 3.2.6 K-means

K-means clustering is a distance-based unsupervised classification method used to partition samples into K groups according to their attribute similarity (Ahmed et al., 2020). In this study, the K-means algorithm was applied to identify structural patterns within the selected multidimensional variables. The algorithm begins by randomly generating K initial cluster centroids, after which each sample is assigned to the nearest centroid based on Euclidean distance. The centroid of each cluster is then updated as the mean vector of all samples within that cluster (Equation 9):


$$\mu_{k}=\frac{1}{|C_{k}|}\sum_{x_{i}\in C_{k}}\;x_{i}$$ (9)

where $\mu_{k}$ is the centroid of cluster k , and $C_{k}$ represents the set of samples assigned to that clusters. The procedure iterates between sample reassignment and centroid updating, aiming to minimize the within-cluster sum of squared errors (SSE) (Equation 10):


$$\mathrm{SSE}=\sum_{k=1}^{K}\;\sum_{x_{i}\in C_{k}}∥x_{i}-\mu_{k}{∥}^{2}$$ (10)

The algorithm terminates when cluster assignments stabilize or a convergence criterion is met. To eliminate scale effects among variables, all input data were standardized before clustering. The optimal number of clusters (K) was determined using both the elbow method and silhouette analysis. The elbow method was used to identify the point at which the reduction in inertia began to level off, while the silhouette score was used to evaluate cluster compactness and separation. The final value of K was selected based on the combined evidence from both criteria.

## 4 Results

### 4.1 Spatial distribution characteristics of traditional villages in China and major tourist cities

#### 4.1.1 Overview of the spatial distribution of traditional villages

**Figure 3:**
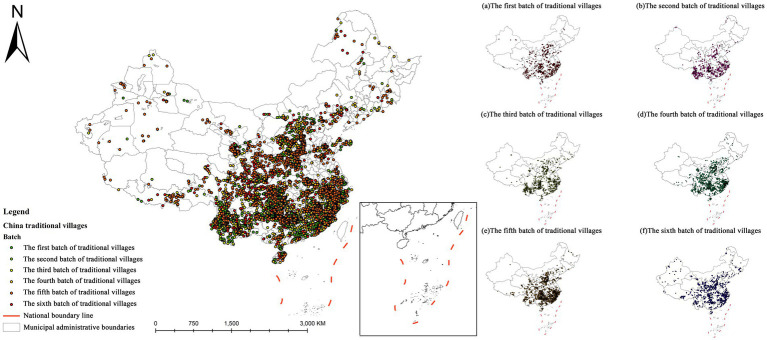
Spatial distribution of traditional villages in China. The main panel presents the overall distribution of all nationally designated traditional villages. Panels **(a–f)** show the spatial distributions of the first, second, third, fourth, fifth, and sixth batches of nationally designated traditional villages, respectively.

The spatial distribution of China’s 8,157 nationally designated traditional villages (Figure 3, Table 1) exhibits a clear geographical imbalance, with a dominant “south-dense, north-sparse” pattern. Traditional villages are highly concentrated in provinces such as Yunnan (777), Guizhou (757), Hunan (704), and Zhejiang (701), which are largely mountainous or hilly regions with long-standing agricultural traditions, relatively limited large-scale urban expansion, and strong continuity of vernacular settlement forms. By contrast, municipalities including Shanghai (5) and Tianjin (8), as well as Northern provinces such as Heilongjiang (26) and Jilin (23), contain far fewer traditional villages, reflecting both the more intensive impacts of modern urbanization and historically weaker settlement continuity in high-latitude or frontier areas. Spatial visualization further identifies five major agglomeration zones of traditional villages: the Shanxi–Hebei–Henan border region, the southeastern coastal belt (Anhui–Fujian–Zhejiang), the Hunan–Guangxi–Guizhou junction, the Yunnan border region, and the Qinghai–Gansu transitional zone. These clusters broadly coincide with areas that historically combined favourable topographic conditions, access to river systems, and relatively well-preserved socio-cultural landscapes.

**Table 1:** Statistics of traditional villages by province.

Province	1st batch	2nd batch	3rd batch	4th batch	5th batch	6th batch	Total
Anhui	25	40	46	52	237	70	470
Beijing	9	4	3	5	1	4	26
Fujian	48	25	52	104	265	58	552
Gansu	7	6	2	21	18	54	108
Guangdong	40	51	35	34	103	29	292
Guangxi Zhuang Autonomous Region (GZAR)	39	30	20	72	119	62	342
Guizhou	90	202	134	119	179	33	757
Hainan	7	0	12	28	17	12	76
Hebei	32	7	18	88	61	70	276
Henan	16	46	37	25	81	70	275
Heilongjiang	2	1	2	1	8	12	26
Hubei	28	15	46	29	88	64	270
Hunan	30	42	19	166	401	46	704
Jilin	0	2	4	3	2	12	23
Jiangsu	3	13	10	2	5	46	79
Jiangxi	33	56	36	50	168	70	413
Liaoning	0	0	8	9	13	15	45
Nei Monggol Autonomous Region	3	5	16	20	2	16	62
Ningxia Hui Autonomous Region	4	0	0	1	1	20	26
Qinghai	13	7	21	38	44	60	183
Shandong	10	6	21	38	50	43	168
Shanxi	48	22	59	150	271	69	619
Shaanxi	5	8	17	41	42	66	179
Shanghai	5	0	0	0	0	0	5
Sichuan	20	42	22	141	108	63	396
Tianjin	1	0	0	2	1	4	8
Xizang Autonomous Region	5	1	5	8	16	45	80
Xinjiang Uygur Autonomous Region	4	3	8	2	1	35	53
Yunnan	62	232	208	113	93	69	777
Zhejiang	43	47	86	225	235	65	701
Chongqing	14	2	47	11	36	54	164

Across the six official batches of designated villages, the cumulative pattern shows both stability and adjustment. Guizhou, Yunnan, Hunan, and Zhejiang consistently rank among the top provinces in each batch, indicating persistent advantages in cultural heritage richness and policy attention. At the same time, the marked increase in the number of fifth- and sixth-batch villages in provinces such as Anhui, Jiangxi, and Guangxi suggests an expanding recognition of traditional settlement value and strengthened heritage identification and protection mechanisms in recent years.

The distribution of traditional villages across China’s major tourist cities further reinforces the pronounced spatial heterogeneity observed at the national scale (Table 2). Only a small number of eastern and southwestern cities such as Hangzhou (63), Chongqing (164), Suzhou (31), Kunming (20), Haikou (16) and Xining (13) host substantial concentrations of traditional villages, reflecting favourable combinations of long-standing agricultural traditions, rich cultural landscapes and relatively constrained historical urban expansion. Cities in the lower Yangtze region, represented by Hangzhou and Suzhou, have benefited from dense water networks, prosperous rural economies and strong heritage protection, while mountainous hubs such as Chongqing and Kunming have preserved village clusters partly because rugged terrain limited large-scale land conversion and maintained distinctive ethnic and architectural traditions. A second group of cities, including Guangzhou, Zhengzhou, Chengdu, Nanning and several provincial capitals, contains moderate numbers of traditional villages and typically occupies transitional zones between core cultural regions and rapidly modernising areas, where heritage continuity coexists with growing urban encroachment and more recent recognition through later designation batches.

**Table 2:** Statistics of traditional villages in major tourist cities of China.

Region	Major tourist cities	1st batch	2nd batch	3rd batch	4th batch	5th batch	6th batch	Total
Northeast	Harbin	0	0	1	0	0	0	1
	Changchun	0	0	0	0	0	3	3
	Dalian	0	0	0	0	0	2	2
East	Beijing	9	4	3	5	1	4	26
	Tianjin	1	0	0	2	1	4	8
	Shijiazhuang	8	3	0	27	15	7	60
	Jinan	1	0	1	4	4	12	22
	Nanjing	0	2	0	0	0	3	5
	Suzhou	2	5	5	2	0	17	31
	Shanghai	5	0	0	0	0	0	5
	Hangzhou	3	5	7	19	16	13	63
	Nanchang	3	5	1	2	2	1	14
	Fuzhou	2	0	5	8	32	4	51
	Shenzhen	1	0	0	0	0	0	1
	Guangzhou	1	8	3	0	1	0	13
	Haikou	3	0	1	9	2	1	16
	Sanya	1	0	0	0	0	0	1
Central	Hohhot	0	2	2	2	0	0	6
	Taiyuan	1	0	1	1	1	0	4
	Zhengzhou	0	0	2	2	4	4	12
	Hefei	0	0	1	0	3	1	5
	Wuhan	2	0	0	2	0	0	4
	Changsha	0	1	0	0	2	0	3
	Nanning	1	3	0	0	6	0	10
West	Urumqi	0	0	0	0	0	0	0
	Lhasa	0	1	2	0	1	1	5
	Lanzhou	3	0	0	1	0	3	7
	Yinchuan	0	0	0	0	0	1	1
	Xining	0	0	0	7	2	4	13
	Xi’an	0	0	0	2	0	3	5
	Chengdu	1	1	0	4	4	0	10
	Chongqing	14	2	47	11	36	54	164
	Guiyang	3	0	0	0	4	0	7
	Kunming	0	7	13	0	0	0	20

The number of traditional villages in major tourist cities generally represents those located within the defined urban tourism study areas. However, for municipalities directly under the central government (Beijing, Shanghai, Tianjin, and Chongqing), the statistics are calculated based on the full administrative area to ensure consistency with provincial-level data and avoid underestimation due to extensive rural territories.

For municipalities such as Chongqing and Beijing, the reported values reflect the full administrative scope, resulting in higher counts compared to other cities. This ensures comparability with provincial-level statistics and avoids distortions caused by differences in spatial delineation.

By contrast, many northern and highly urbanised metropolitan areas contain relatively few officially designated traditional villages. Dalian, Changchun, Harbin, Yinchuan, and Urumqi record only a handful of villages or none at all, while Shanghai contains only five traditional villages. Fuzhou, although located in the developed coastal region, still retains 51 officially designated traditional villages, reflecting the persistence of traditional settlement heritage in parts of southeastern China despite rapid urban development. Ethnic minority regions present a more mixed picture: cities such as Lhasa, Hohhot, Kunming and Guiyang retain smaller but culturally significant clusters of villages, often with a higher proportion of later-batch designations, suggesting that the recognition of minority settlement heritage has strengthened in recent years. Overall, the city-level distribution confirms that traditional villages are unevenly embedded within major tourist destinations, shaped jointly by historical settlement trajectories, physical geography and differentiated urban development pathways.

Based on the traditional village counts of major tourist cities (Table 2), the coefficient of variation (CV) was used to further evaluate the inter-city disparity for each batch as well as for the total number of traditional villages. The results show that the CV values for the six batches are 1.59 (1st batch), 1.69 (2nd batch), 2.14 (3rd batch), 1.95 (4th batch), 2.17 (5th batch), and 1.86 (6th batch), respectively, while the CV for the total number of traditional villages across all batches is 1.33. These high CV values indicate pronounced heterogeneity in the distribution of traditional villages among major tourist cities. In particular, the third and fifth batches exhibit the largest coefficients of variation (2.14 and 2.17), implying that traditional villages identified in these stages are highly concentrated in a small number of cities such as Hangzhou, Suzhou, Chongqing, and Kunming, whereas many other tourist cities have very few or no listed villages. By contrast, the CV for the overall total (1.33) is relatively lower, suggesting that, when all six batches are considered jointly, the spatial imbalance among major tourist cities is somewhat alleviated, and the coverage of traditional villages in the national tourism city system has become more extensive.

It should be noted that the number of traditional villages reported at the provincial level (Table 1) differs from the city-level statistics (Table 2) due to differences in spatial scope. Provincial totals include all villages within the entire administrative boundary, whereas city-level counts only refer to villages located within the selected major tourist cities. For municipalities such as Chongqing, which contain extensive rural counties beyond the core urban tourism areas, this distinction leads to noticeable differences in reported values.

#### 4.1.2 Spatial equity characteristics of traditional village distribution

Using the Nearest Neighbor tool in GIS, the average nearest distance and Nearest Neighbor Index (NNI) were calculated for traditional villages across all provinces (Table 3). The results reveal clear spatial heterogeneity and distinct clustering characteristics at the national scale. Overall, most provinces exhibit NNI values less than 1, indicating that traditional villages in China generally present a clustered spatial pattern rather than a random or evenly dispersed distribution. Provinces with dense historical settlements, mountainous terrain, or preserved cultural landscapes tend to show particularly strong clustering.

**Table 3:** Average nearest neighbor index of traditional villages in each province.

Province	Average nearest distance (KM)	Nearest index	Province	Average nearest distance (KM)	Nearest index
Anhui	0.4626	0.5691	Liaoning	2.2506	0.7603
Beijing	0.7605	0.8323	Nei Monggol Autonomous Region	4.3024	0.4972
Fujian	0.6047	0.6968	Ningxia Hui Autonomous Region	1.9494	0.8493
Gansu	2.0246	0.5497	Qinhai	0.4359	0.2373
Guangdong	0.9342	0.5774	Shandong	0.8551	0.5519
Guangxi Zhuang Autonomous Region (GZAR)	0.8048	0.4886	Shanxi	0.565	0.5227
Guizhou	0.5286	0.6183	Shaanxi	1.4036	0.6615
Hainan	0.8527	0.7731	Shanghai	2.2751	2.2273
Hebei	0.5772	0.4064	Sichuan	1.308	0.5983
Henan	0.8113	0.5493	Tianjin	2.0617	1.8071
Heilongjiang	4.5317	0.7829	Xizang Autonomous Region	3.14	0.5925
Hubei	0.9049	0.5321	Xinjiang Uygur Autonomous Region	5.194	0.6496
Hunan	0.6197	0.6431	Yunnan	0.791	0.5652
Jilin	3.2073	0.9101	Zhejiang	0.494	0.6606
Jiangsu	1.4689	0.7602	Chongqing	1.0282	0.6576
Jiangxi	0.706	0.5857			

Several southeastern and southwestern provinces demonstrate the highest degree of clustering, with notably low average nearest distances and small NNI values. For example, Guangxi (NNI = 0.4886), Guizhou (0.6183), Fujian (0.6968), Hunan (0.6431), and Zhejiang (0.6606) display highly compact spatial arrangements. These regions are known for their complex terrain, multi-ethnic settlement patterns, and long-standing agricultural traditions, all of which contributed to the formation of densely distributed village clusters. Similarly, Shanxi (0.5227) and Hebei (0.4064) also show strong clustering due to well-preserved traditional courtyard settlements and historically stable rural populations.

In contrast, northern and northwestern provinces present markedly larger nearest distances and higher NNI values, indicating weaker clustering or more dispersed distributions. Provinces such as Xinjiang (average distance = 5.19 km), Heilongjiang (4.53 km), Inner Mongolia (4.30 km), Jilin (3.21 km), and Tibet (3.14 km) exhibit the most dispersed village patterns. These areas feature vast land areas, sparse populations, and unique nomadic or semi-nomadic settlement histories, which inherently limit the formation of compact village clusters.

A small number of provinces show NNI values approaching or exceeding 1, suggesting random or dispersed distributions. Notably, Shanghai (NNI = 2.2273) and Tianjin (1.8071) present the highest dispersion, largely due to their high urbanization levels and the limited number of surviving traditional villages. Beijing (0.8323) and Ningxia (0.8493) also show weaker clustering, reflecting a combination of intensive urban expansion and historically limited rural settlement density.

Based on the provincial distribution of traditional villages (Table 1), the geographical concentration indices were calculated for all six batches. The results show that the concentration index of the first batch is 25.61, followed by 36.66 for the second batch, 29.94 for the third batch, 26.96 for the fourth batch, 27.76 for the fifth batch, and 20.50 for the sixth batch. These values indicate that the degree of spatial clustering at the provincial scale varies substantially across batches. The second batch exhibits the highest concentration index, suggesting that its traditional villages were predominantly concentrated in a small number of provinces with rich cultural resources and well-established preservation bases, resulting in a pronounced spatial imbalance. The third to fifth batches present moderate concentration levels, reflecting a transitional phase in which the distribution of traditional villages gradually shifted from strong clustering toward wider regional dispersion. In contrast, the sixth batch shows the lowest concentration index, implying that the most recent identification round is characterized by a more balanced pattern, with expanded coverage and reduced interprovincial disparities.

Similarly, inequality indices were calculated for each batch to further assess the degree of interprovincial disparity. The results reveal that the inequality index of the first batch is 0.5314, followed by 0.7014 for the second batch, 0.5877 for the third batch, 0.5785 for the fourth batch, 0.6041 for the fifth batch, and 0.3093 for the sixth batch. These findings demonstrate significant differences in the spatial distribution of traditional villages across batches, showing an overall evolution of “high inequality in the early stages, gradually shifting toward greater balance in later stages.” The second batch records the highest inequality index, indicating that its traditional villages were highly concentrated in a few culturally resource-rich provinces, with the most pronounced interprovincial disparities. The third to fifth batches exhibit moderate inequality levels, suggesting an expanding identification scope yet still notable provincial differences. The sixth batch, with the lowest inequality index of 0.3093, reflects the most balanced spatial distribution, indicating that a wider range of provinces has begun to meet the criteria for traditional village designation.

### 4.2 Correlation between traditional villages and tourism development levels in major tourist cities

To further investigate the relationship between traditional villages and tourism development, Pearson’s product–moment correlation and Spearman’s rank correlation were calculated between the number of traditional villages in each major tourist city and five tourism indicators. The Pearson coefficients indicate weak to moderate positive linear associations with domestic tourism volume and domestic tourism income, with r =0.28 for domestic tourist arrivals (X2) and r =0.32 for domestic tourism income (X4). By contrast, the correlation with inbound tourist arrivals (X1) is very weak ( r =0.17), and the association with international tourism foreign-exchange earnings (X3) is slightly negative ( r = − 0.09). The share of tourism income in GDP (X5) shows almost no linear relationship with the number of traditional villages ( r =0.01). Spearman’s rank correlations display a similar pattern, with negligible or very weak monotonic relationships for X1–X3 ( ρ ranging from −0.10 to 0.03) and only weak positive correlations with domestic tourism income (X4, ρ=0.21 ) and the tourism-income-to-GDP ratio (X5, ρ=0.23 ). Overall, these results suggest that cities with more traditional villages tend to exhibit somewhat higher levels of domestic tourism demand and income, yet the strength of these associations remains modest and is not statistically significant at the 5% level, implying that traditional villages constitute only one of several factors contributing to urban tourism performance.

### 4.3 Analysis of the correlation between traditional village and tangible spatial carriers

Traditional villages, as key players in rural tourism, play a crucial role in preserving regional historical and cultural legacies while stimulating local economic growth and cultural preservation. These villages offer tourists unique experiences such as folk activities, traditional crafts, and local cuisine, attracting those seeking authentic cultural experiences. Engaging in tourism helps these villages generate economic benefits and preserve cultural heritage, facilitating exchanges between urban and rural areas and contributing to urbanization. This paper explores the relationship between traditional villages and local tourism resources to understand how they enhance tourist appeal and drive regional economic and urban development. China has officially recognized 8,157 traditional villages in six batches, distributed unevenly across the country, with the highest concentrations in Yunnan, Guizhou, Hunan, Zhejiang, and Shanxi provinces. In this study, a sample refers to a 20 km × 20 km grid cell generated by the spatial relationship model. Each sample records the presence or absence of traditional villages and the corresponding cultural–tourism carrier.

#### 4.3.1 Traditional village and A-level scenic spots

As primary drivers of tourism, urbanization, and economic growth, A-level scenic spots are crucial for enhancing the attractiveness of tourism cities by providing unique travel experiences and promoting local economic and infrastructural development. As of 2023, China boasts 14,900 A-level tourist locations. This study utilized a spatial relationship model to analyze the correlation between traditional villages and A-level tourist spots, identifying 304 grid samples containing both traditional villages and A-level scenic spots, 203 with only traditional villages, and 831 with only A-level tourist spots. The spatial correlation index R was calculated at 0.837, indicating a significant positive correlation between the spatial distribution of traditional villages and A-level tourist spots in major tourism cities. Of the mixed samples, there are 812 A-level scenic spots, representing 5.45% of the national total, including 26 5A-level spots, accounting for 7.74% of the total number of 5A-level spots. These 5A-level spots, the highest standard in China’s tourism industry, are pivotal in promoting rural tourism and urbanization by providing high-quality services and cultural experiences, thereby enhancing the local tourism brand and economic development in surrounding rural areas. Surrounding villages often form tourism clusters centered on these 5A-level spots, leveraging their unique landscapes and cultural heritage to develop homestays, agritourism, and handicraft industries, thus improving rural income and living standards. The sample also includes 314 4A-level, 365 3A-level, 105 2A-level, and 2 1A-level scenic spots, representing 6.99, 4.64, 5.08, and 2.22% of their respective categories nationwide.

**Figure 4:**
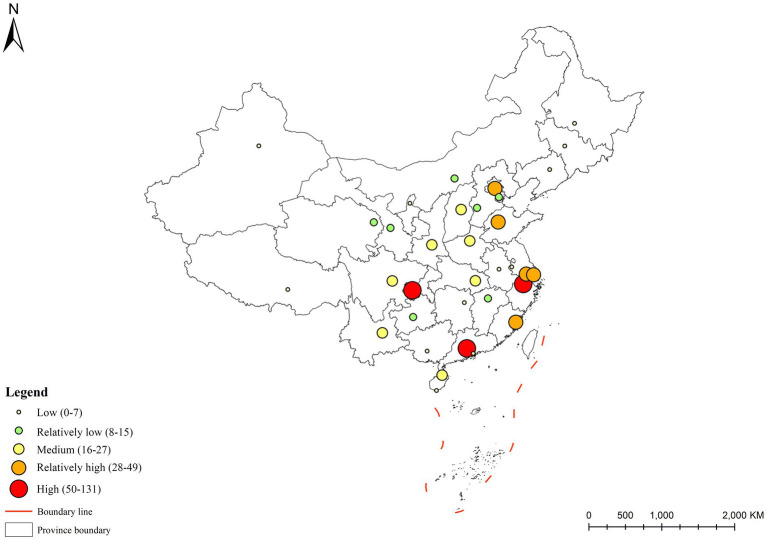
Number of A-level scenic spots located in all levels of major tourist cities.

The number of tourist spots within samples containing both traditional villages and A-level scenic spots across 34 major tourist cities were categorized into five levels using the natural breaks method in ArcGIS. The categories were as follows: high, relatively high, medium, relatively low, and low. The results are displayed in Figure 4. In the samples containing both traditional villages and A-level scenic spots, cities classified with a high number of A-level attractions include Hangzhou, Chongqing, and Guangzhou, with 131, 95, and 95 attractions, respectively. The primary reason for this is that these cities have a significant number of both traditional villages and A-level tourist spots, with a high degree of spatial correlation between the two, indicating a greater potential for integrated development compared to other cities. In samples that also include traditional villages, cities with a relatively high number of A-level tourist spots include Jinan, Fuzhou, Beijing, Suzhou, and Shanghai, all of which are located in the eastern coastal region. Cities that have been classified as having a medium number of A-level scenic spots include Wuhan, Chengdu, Xi’an, Zhengzhou, Taiyuan, Kunming, and Haikou. These cities are primarily located in the central and western regions of the country. The majority of these cities have become significant tourist destinations due to their distinctive geographical locations and rich historical and cultural heritage. Nevertheless, cities with a relatively low number of A-level scenic spots are distributed primarily in more remote areas of southwest, central China, and northwest China, with a total of seven and twelve cities, respectively, falling into this category.

It should be noted, however, that the number of traditional villages within these city boundaries varies substantially and is generally modest even among cities classified at higher tiers. Cities in the high category (Hangzhou, Chongqing, and Guangzhou) contain 63, 164, and 13 traditional villages, respectively, within their administrative boundaries (Table 2), which already represent the upper end of the distribution among major tourist cities. Cities in the relatively high and medium categories host between 4 and 60 traditional villages, while many cities in the lower tiers contain fewer than 10 or even none. This pattern reflects the broader distribution reality discussed in Section 3.1: the majority of nationally designated traditional villages are located in mountainous and rural areas characterised by low levels of urbanisation, even within economically developed provinces. The spatial correlation with A-level scenic spots therefore captures structural adjacency within regional tourism systems rather than direct physical co-location within urbanised city cores.

#### 4.3.2 Traditional village and intangible cultural heritage

Intangible cultural heritage (ICH) is a vital aspect of traditional village culture, encompassing traditional skills, folklore, languages, music, dances, unique lifestyles, festival activities, and local customs, all of which contribute significantly to cultural heritage and social identity. Integrating ICH with tourism enhances the attractiveness and economic vitality of rural areas like traditional villages, protects intangible cultural heritage, and advances urban tourism and urbanization. Recently, local knowledge, folk art, and traditional customs have gained recognition for their role in promoting rural and urban tourism development, becoming key tourism resources within traditional villages. This study applied a spatial relationship model to explore the spatial correlation between traditional villages and ICH, identifying 43 samples with both elements, 464 samples containing only traditional villages, and 190 containing only ICH. The spatial correlation index R calculated was 0.0014, indicating a non-significant correlation at the 5% level, suggesting a low degree of spatial correlation among the three tangible spatial carriers examined, possibly due to the random geographical distribution of traditional villages and ICH or varying management and protection measures. The samples including both traditional villages and ICH contain only 170 ICH elements, just 4.71% of the national total, highlighting a low potential for integrated development due to the impacts of modern urban culture and insufficient management and protection, leading to the gradual loss of traditional buildings, lifestyles, and cultural customs.

**Figure 5:**
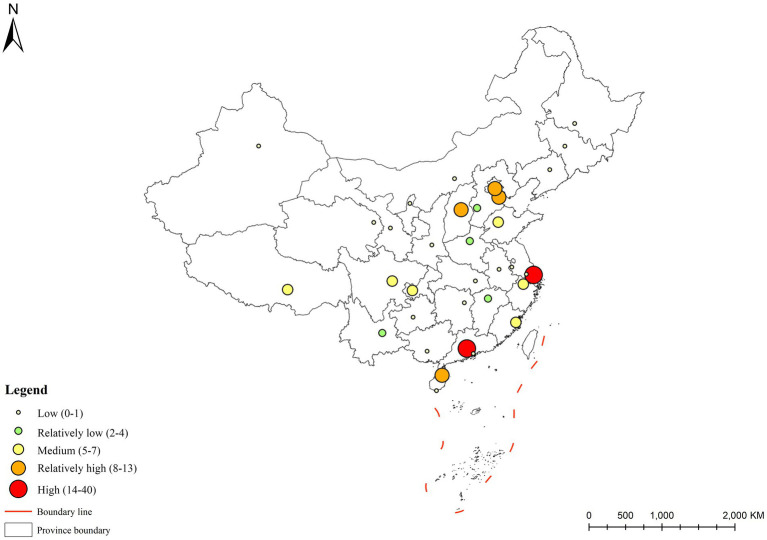
Number of ICH located in all levels of major tourist cities.

Using ArcGIS and the natural breaks method, intangible cultural heritage (ICH) items in 34 major tourist cities were categorized into five levels based on their presence in traditional villages, as shown in Figure 5. Cities with a high level of ICH items include Guangzhou and Shanghai, which have 40 and 37 ICH items, respectively. Guangdong, where Guangzhou is located, ranks among the top five provinces with 164 ICH items. Despite having fewer ICH items, Shanghai’s effective urban cultural heritage management and strong cultural promotion capabilities foster close integration between traditional villages and ICH, suggesting a high potential for integrated development. Cities like Haikou, Taiyuan, Tianjin, and Beijing also exhibit relatively high ICH levels, ranging from 9 to 13 items each, and are located in culturally rich regions like the Beijing-Tianjin-Hebei area. The study reveals that the integration and spatial association between traditional villages and ICH in most major tourist cities are relatively low. Although traditional villages, rich in historical culture, should ideally promote and complement ICH, in practice, they often develop almost independently. Effective protection and development of ICH are essential for boosting rural tourism and enhancing rural revitalization. Many rural tourism destinations now integrate ICH elements such as handicrafts and folk performances into their offerings, enhancing tourist experiences, generating economic benefits, and fostering cultural protection, thus creating a beneficial cycle of culture and tourism promotion.

#### 4.3.3 Traditional village and key cultural relics protection building

Under the guidelines of the State Council, various sites are recognized as integral parts of China’s national cultural heritage. These immovable cultural relics, valued for their historical, artistic, and scientific significance, include ancient sites, tombs, buildings, cave carvings, important modern historical sites, and representative structures. Despite the rapid urbanization that has incorporated many traditional villages into urban areas, several villages with unique characteristics and historical importance have been designated as key cultural heritage sites. These sites are not only popular scenic spots but also venues for educational and exhibition activities aimed at promoting public awareness of traditional history and culture. To analyze the spatial correlation between traditional villages and key cultural heritage sites, this study used a spatial relationship model. It identified 141 samples containing both traditional villages and key cultural heritage sites, 366 samples with only traditional villages, and 320 with only key cultural heritage sites. The spatial correlation index R was calculated at 0.1421, which passed the 5% significance level test, confirming a significant positive spatial correlation and a high degree of spatial association in major tourist cities. Among these samples, there were 283 key cultural heritage sites, representing 5.59% of the national total, indicating that the majority of key cultural heritage sites are derived from sources other than traditional villages.

**Figure 6:**
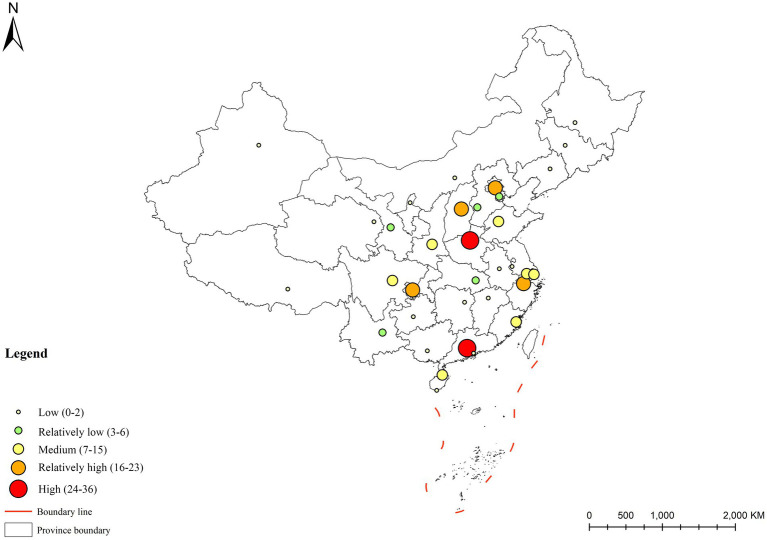
Number of key cultural relics protection building located in all level of major tourist cities.

Based on the number of key cultural relics protection building in the samples that contained both traditional villages and heritage sites, the results were categorized into five levels: high, relatively high, medium, relatively low, and low, as shown in Figure 6. Cities with a high number of sites included Zhengzhou and Guangzhou, while cities with a relatively high number were Taiyuan, Chongqing, and Beijing. Cities with a medium number included Xi’an, Fuzhou, Haikou, Suzhou, Jinan, Shanghai, and Chengdu. Of the twelve cities in the high, relatively high, and medium tiers, seven were situated in the eastern region, two in the central region, and three in the western region. These findings suggest the existence of notable regional disparities in the integration of traditional villages and key cultural heritage sites throughout China. The eastern region, which exhibits greater economic advancement and a more substantial repository of cultural resources, also allocates a greater proportion of its resources to the preservation of historical culture. In contrast, historical and cultural cities in the central and western regions, such as Xi’an and Taiyuan, are able to draw upon a wealth of historical resources and benefit from the implementation of focused cultural preservation strategies.

### 4.4 Classification of traditional village and rural tourism development types

**Figure 7:**
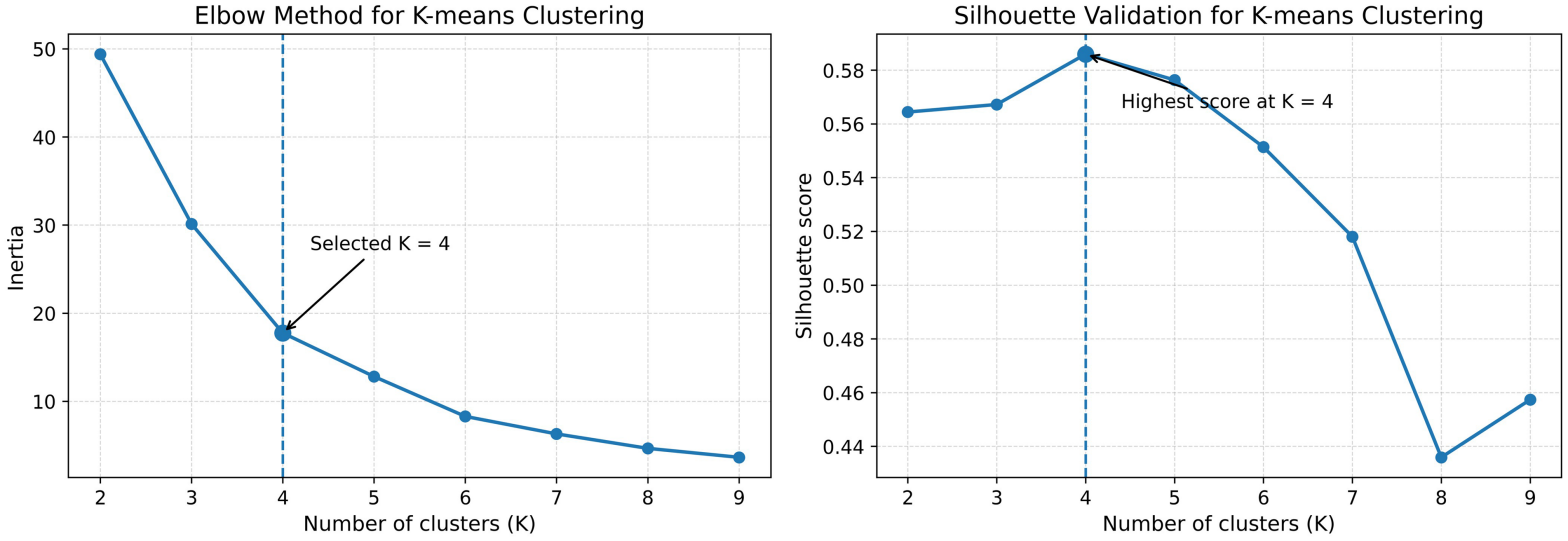
Graph of K values and inertia.

K-means clustering was applied to analyze the number of A-level scenic spots, intangible cultural heritage, and key cultural heritage sites in samples that include both traditional villages and tangible carriers, in order to classify the traditional villages and tourism integration development areas with similar advantages. Five clustering models were set, with K values of 2, 3, 4, 5, and 6. The elbow method was used to determine the optimal K value. The elbow method shows that the reduction in inertia becomes substantially smaller after K = 4. In addition, the silhouette score reaches its highest value at K = 4, indicating that the four-cluster solution provides the best balance between within-cluster compactness and between-cluster separation. Therefore, K = 4 was selected for the final typology (see Figure 7).

The mean numbers of A-level scenic spots, intangible cultural heritage items, and key cultural heritage sites contained in samples that include both traditional villages and tangible carriers exhibit notable differences. Consequently, the 34 major tourist cities in China can be classified into four types, as illustrated in Table 4.

**Table 4:** Traditional village and tourism integration development type division.

Type	A-level scenic spots (average)	ICH (average)	Key cultural relics protection building (average)	Cities
Class I resource-rich integration area	65	29	23	Guangzhou, Shanghai
Class II scenic-village Integration area	113	6	22	Chongqing, Hangzhou
Class III balanced development area	32	6	17	Zhengzhou, Taiyuan, Beijing, Xi’an, Fuzhou, Haikou, Suzhou, Jinan, Chengdu
Class IV potential development area	9	1	2	Shijiazhuang, Wuhan, Kunming, Lanzhou, Tianjin, Nanchang, Hohhot, Nanning, Guiyang, Lhasa, Xining, Nanjing, Shenzhen, Dalian, Hefei, Changsha, Sanya, Changchun, Haerbin, Yinchuan, Urumqi

Category I comprises Guangzhou and Shanghai, designated as Resource-Rich Integration Areas. These cities are distinguished not by the absolute number of traditional villages they contain, but by the exceptionally high density of A-level scenic spots, intangible cultural heritage items, and key cultural heritage sites co-occurring with their existing traditional villages. This pattern indicates that the traditional villages present in these cities are embedded within the richest concentration of cultural–tourism carriers among all cities studied, reflecting a high degree of integration quality. Accordingly, this category represents cities where existing traditional villages are most thoroughly surrounded by diversified tourism resources, suggesting strong structural conditions for heritage–tourism synergy.

Category II includes Chongqing and Hangzhou, where the average numbers of A-level scenic spots and key cultural heritage sites in co-occurrence samples are high, but the number of intangible cultural heritage items is relatively low compared to Category I. This category is characterised by a development advantage in integrating traditional villages with scenic and tangible heritage resources, with considerable untapped potential for further incorporating intangible cultural heritage to enrich rural tourism offerings.

Category III encompasses nine cities, including Zhengzhou, Taiyuan, and Beijing. These cities exhibit moderate average values across all three carrier types, falling neither at the maximum nor the minimum. They represent transitional integration areas where traditional villages coexist with a meaningful but unevenly developed tourism resource base, and where each carrier type retains significant potential to drive future traditional village tourism development.

Category IV comprises 20 major tourist cities, which record the lowest average numbers of A-level scenic spots, intangible cultural heritage items, and key cultural heritage sites in co-occurrence samples. These cities are categorised as low-carrier integration areas, where the limited density of surrounding tourism resources constrains the immediate potential for integrated heritage–tourism development, and where foundational investment in cultural infrastructure and resource activation is most needed.

## 5 Discussion

The findings of this study can be further interpreted in relation to existing research on rural heritage and tourism integration. Previous studies have emphasized the role of traditional villages as important carriers of cultural resources and drivers of rural tourism development (Yanan et al., 2024; Li et al., 2023), often suggesting a positive relationship between heritage resources and tourism performance. However, most of these studies are based on case studies or regional analyses that tend to highlight localized success stories. By contrast, the national-scale results presented here reveal that the relationship between traditional villages and overall urban tourism performance is relatively weak, suggesting that their contribution to tourism development is more context-dependent and spatially uneven than previously assumed, thereby refining existing understandings.

More broadly, the results indicate that the spatial differentiation of traditional villages in China is shaped by intertwined historical, geomorphological, socio-economic, and policy factors, yet this differentiation is only weakly reflected in city-level tourism performance. Dense clusters in southeastern and southwestern regions coincide with long-standing agricultural settlement patterns and strong cultural continuity, whereas northern and coastal metropolitan areas retain relatively few traditional villages due to intensive urbanisation. At the city scale, the limited number of traditional villages and their weak correlation with aggregate tourism indicators suggest that they remain marginal to mainstream, city-centred tourism systems. At the same time, stronger associations with A-level scenic spots and protected cultural relics, combined with minimal linkage to intangible cultural heritage, indicate that traditional villages are more closely embedded in networks of tangible heritage than in practice-based cultural systems. The fourfold typology further demonstrates that integration potential is highly uneven, ranging from resource-rich clusters to low-capacity areas where both tourism and heritage bases are limited.

These findings point to a fundamental distinction between urban tourism systems and rural tourism networks. Although this study adopts major tourist cities as analytical units, traditional villages are spatially concentrated in less urbanized, peripheral regions, often beyond the core functional areas of these cities. This distributional mismatch is particularly evident in economically developed but topographically diverse provinces such as Zhejiang, Fujian, and Guangdong, where traditional villages remain concentrated in inland mountain areas despite the high overall urbanisation levels of their provincial capitals. As a result, their integration into tourism systems tends to occur through indirect, network-based linkages rather than direct incorporation into urban tourism economies. From this perspective, traditional villages may be understood, at the spatial pattern level, as nodes within interconnected urban–rural tourism networks, where A-level scenic spots and other cultural resources function as intermediary connectors. This multi-scalar interpretation helps explain why strong spatial associations do not necessarily translate into strong correlations with urban tourism performance.

The mechanisms through which resource richness may drive traditional village tourism development can be understood through the concept of agglomeration and spillover effects within regional tourism systems. A-level scenic spots, particularly those at the 4A and 5A levels, function as anchor attractions that generate substantial visitor flows and establish destination awareness. Traditional villages situated within the geographic catchment of these anchor attractions are well positioned to capture secondary visitor interest, benefiting from the infrastructure investment, transport accessibility, and marketing resources that accompany high-grade scenic designations. In this sense, the density of A-level scenic spots co-occurring with traditional villages reflects not merely statistical adjacency, but a structural proximity to established tourism demand that may lower the threshold for village-level tourism activation. Similarly, the co-presence of key cultural relics enriches the interpretive depth of a destination, enabling the construction of integrated heritage routes that position traditional villages as complementary nodes rather than isolated attractions. The comparatively weak association with intangible cultural heritage, however, suggests that practice-based and performative cultural resources remain less systematically embedded in these tourism networks, representing an underdeveloped but potentially high-value dimension of integration.

Regarding the role of transportation in mediating integration, the present analysis highlights a structural tension that is particularly consequential for traditional villages. As demonstrated in Section 3.1, the majority of nationally designated traditional villages are concentrated in mountainous and topographically complex regions including Guizhou, Yunnan, Hunan, and parts of Fujian and Zhejiang where road connectivity and inter-modal accessibility have historically been limited. While proximity to A-level scenic spots may indicate potential for tourism spillover, this potential can only be realised when transport infrastructure enables visitor movement between urban gateways and rural village settings. Cities with well-developed transport hubs including high-speed rail termini, regional airports, and expressway networks are better positioned to facilitate this movement and integrate surrounding traditional villages into functional tourism circuits. By contrast, in cities where traditional villages are accessible only via rural or mountainous roads, the spatial correlation with scenic resources may not translate into actual visitor flows. This transport-heritage mismatch represents a key structural constraint on village-level tourism integration and helps explain why cities with comparatively high traditional village counts do not always exhibit stronger associations with urban tourism performance indicators, as observed in the correlation analysis in Section 3.2. Future research incorporating transport accessibility data at the village level would allow this constraint to be more directly quantified.

The spatial heterogeneity identified in this study is reflected across multiple scales and dimensions. At the national scale, the pronounced “south-dense, north-sparse” distributional pattern documented in Section 3.1 establishes a foundational geographic unevenness. Clustering indices confirm strong agglomeration in southeastern and southwestern provinces, a pattern that permeates all subsequent analyses that permeates all subsequent analyses. At the city scale, the coefficient of variation across major tourist cities (CV = 1.33 for total village counts) confirms that even within the selected urban tourism system, traditional village resources are highly unequally distributed, with a small number of cities such as Chongqing and Hangzhou accounting for a disproportionate share of village counts. At the integration scale, the four-class typology reveals that heterogeneity is not simply a function of village quantity: cities in Class I hold very few traditional villages yet exhibit the highest carrier density per co-occurrence sample, while cities in Class IV contain few of both villages and surrounding resources. This multi-scalar structure indicates that spatial heterogeneity in traditional village tourism is produced by the simultaneous interaction of settlement history, geomorphological constraints, urbanisation trajectories, and the uneven geographic distribution of institutionally recognised cultural resources and that any single metric is insufficient to capture its full complexity.

Importantly, traditional villages are spatially positioned in ways that suggest the possibility of stronger functional linkages within rural tourism systems, where localized cultural landscapes, ecological resources, and community-based activities including agritourism, village-based homestay economies, and intangible heritage performances may provide more direct and organic support for their development and revitalization though this remains to be verified through direct examination of tourism flows and community-level data. The city-level analytical framework adopted in this study captures structural adjacency between village heritage and urban tourism infrastructure, but does not fully represent the ground-level dynamics through which traditional villages actually attract visitors, generate rural income, and support community livelihoods. This suggests that the role of traditional villages should not be evaluated solely within city-centred tourism frameworks, but rather within broader regional and rural tourism contexts.

Based on the spatial patterns identified, the following planning directions are suggested as hypotheses warranting further investigation rather than as policy prescriptions directly verified by this study. In high-capacity regions, the spatial evidence points to potential benefits from strengthening urban–rural heritage corridors and designing integrated tourism routes that connect villages with major cultural attractions. In areas with moderate resources, improving accessibility and inter-village connectivity can help transform dispersed heritage assets into coherent tourism systems. In low-capacity regions, foundational investments in infrastructure, cultural activation, and local capacity building are essential. Across all contexts, revitalising intangible cultural heritage within village settings remains crucial for reinforcing cultural depth and avoiding purely attraction-oriented development.

Finally, this study is subject to several limitations. Due to data availability constraints, the analysis focuses on A-level scenic spots and major tourist cities as representative indicators of the cultural–tourism system. Future research should directly examine the spatial relationships between traditional villages and rural scenic resources, rural tourism clusters, and county-level tourism systems complementing city-level analysis with ground-level evidence that may reveal stronger and more actionable correlations in direct support of rural revitalization goals. This represents an important direction for extending the current analytical framework.

## 6 Conclusion

This study provides a national-scale assessment of the spatial differentiation of traditional villages in China and their potential integration within cultural–tourism networks. The results reveal pronounced geographical imbalance shaped by historical, geomorphological, socio-economic, and policy factors, with strong clustering in southeastern and southwestern provinces and marked sparsity in northern and coastal metropolitan areas. Although traditional villages show weak association with overall urban tourism performance, they exhibit strong spatial alignment with scenic and tangible heritage resources and a clear mismatch with intangible cultural heritage. The four-class typology further identifies differentiated integration pathways, ranging from high-capacity heritage clusters with strong synergistic potential to low-resource areas requiring foundational cultural activation and infrastructural improvement. These findings provide spatial evidence consistent with the interpretation that traditional villages may function as selectively embedded nodes within broader cultural–tourism spatial systems, though the mechanisms through which this embedding operates require further empirical investigation. The spatial patterns identified suggest several planning directions that merit further consideration: coordinated urban–rural heritage planning, improved accessibility and connectivity, revitalization of intangible cultural practices in rural contexts, and region-specific strategies for integrated tourism development. These directions are grounded in the spatial associations and typological differences identified in this study, and should be understood as hypotheses to be tested rather than conclusions directly warranted by the current analysis. Future research should expand beyond officially listed villages and city-level indicators by incorporating multi-temporal datasets, finer-grained socio-economic variables, and comparative international cases to better understand rural heritage sustainability under uneven development pressures.

## Data Availability

The original contributions presented in the study are included in the article/supplementary material, further inquiries can be directed to the corresponding author.
